# Auditory Memory Decay as Reflected by a New Mismatch Negativity Score Is Associated with Episodic Memory in Older Adults at Risk of Dementia

**DOI:** 10.3389/fnagi.2018.00005

**Published:** 2018-02-02

**Authors:** Daria Laptinskaya, Franka Thurm, Olivia C. Küster, Patrick Fissler, Winfried Schlee, Stephan Kolassa, Christine A. F. von Arnim, Iris-Tatjana Kolassa

**Affiliations:** ^1^Clinical and Biological Psychology, Institute of Psychology and Education, Ulm University, Ulm, Germany; ^2^Department of Psychology, University of Konstanz, Konstanz, Germany; ^3^Faculty of Psychology, TU Dresden, Dresden, Germany; ^4^Department of Neurology, Ulm University, Ulm, Germany; ^5^Department for Psychiatry and Psychotherapy, University Hospital Regensburg, Regensburg, Germany; ^6^SAP (Switzerland) AG, Tägerwilen, Switzerland

**Keywords:** mismatch negativity, auditory memory, cognition, episodic memory, mild cognitive impairment, subjective memory impairment, Alzheimer’s disease, event-related potentials

## Abstract

The auditory mismatch negativity (MMN) is an event-related potential (ERP) peaking about 100–250 ms after the onset of a deviant tone in a sequence of identical (standard) tones. Depending on the interstimulus interval (ISI) between standard and deviant tones, the MMN is suitable to investigate the pre-attentive auditory discrimination ability (short ISIs, ≤ 2 s) as well as the pre-attentive auditory memory trace (long ISIs, >2 s). However, current results regarding the MMN as an index for mild cognitive impairment (MCI) and dementia are mixed, especially after short ISIs: while the majority of studies report positive associations between the MMN and cognition, others fail to find such relationships. To elucidate these so far inconsistent results, we investigated the validity of the MMN as an index for cognitive impairment exploring the associations between different MMN indices and cognitive performance, more specifically with episodic memory performance which is among the most affected cognitive domains in the course of Alzheimer’s dementia (AD), at baseline and at a 5-year-follow-up. We assessed the amplitude of the MMN for short ISI (stimulus onset asynchrony, SOA = 0.05 s) and for long ISI (3 s) in a neuropsychologically well-characterized cohort of older adults at risk of dementia (subjective memory impairment, amnestic and non-amnestic MCI; *n* = 57). Furthermore, we created a novel difference score (ΔMMN), defined as the difference between MMNs to short and to long ISI, as a measure to assess the decay of the auditory memory trace, higher values indicating less decay. ΔMMN and MMN amplitude after long ISI, but not the MMN amplitude after short ISI, was associated with episodic memory at baseline (*β* = 0.38, *p* = 0.003; *β* = −0.27, *p* = 0.047, respectively). ΔMMN, but not the MMN for long ISIs, was positively associated with episodic memory performance at the 5-year-follow-up (*β* = 0.57, *p* = 0.013). The results suggest that the MMN after long ISI might be suitable as an indicator for the decline in episodic memory and indicate ΔMMN as a potential biomarker for memory impairment in older adults at risk of dementia.

## Introduction

The global number of people aged 50 years and older is constantly increasing (e.g., Gerland et al., 2014; United Nations, 2015). Age is the major risk factor of Alzheimer’s dementia (AD). Until 2050 about three million older adults will be affected by AD in Germany (Bickel, 2016) and 132 million worldwide (Prince et al., 2015). Alzheimer’s pathology is characterized by amyloid-beta and tau deposition in the entorhinal cortex, hippocampus, neocortex and other brain regions (for a review see Ballard et al., 2011). Furthermore, Alzheimer’s pathology is associated with deficiencies in neuronal signal transmission and neuronal death and precedes the manifestation of cognitive symptoms by many years or even decades (for a review see Bateman et al., 2012).

Mild cognitive impairment (MCI) can be a prodromal syndrome of AD and is therefore intensively studied in the context of early diagnosis of the disease. Individuals with MCI show cognitive decline in at least one cognitive domain, while overall daily functioning is still intact (Petersen, 2016). MCI is associated with an increased risk of dementia, particularly AD, compared to the general population with a conversion rate to dementia of about 8%–15% per year (Petersen, 2016). Amnestic MCI (aMCI) has a higher progression rate than non-amnestic MCI (naMCI; Petersen, 2016). Furthermore, recent research indicates that subjective memory impairment (SMI) that cannot be confirmed during objective testing is associated with an increased risk for AD up to 6 years later (Jessen et al., 2014).

Event-related potentials (ERPs) can provide further insights into neurophysiological correlates of cognitive decline and neuropathology in old age (e.g., Papaliagkas et al., 2008; Lai et al., 2010; Vecchio and Määttä, 2011; Thurm et al., 2013), with the advantages of being non-invasive and cost-efficient. One of the most widely investigated ERP components in EEG research is the mismatch negativity (MMN; Näätänen et al., 1978). The MMN is elicited when a presentation that has been automatically predicted by the central nervous system is violated (for a review see Näätänen et al., 2011), i.e., when a deviant tone is presented in a sequence of standard tones. It peaks at about 100–250 ms after the onset of the deviant (for a review see e.g., Fishman, 2014). The MMN indicates a generally pre-attentive process, but can be modulated by attention (e.g., Erickson et al., 2016). Previous studies suggest an association between MMN in a passive paradigm and the active deviant tone detection (e.g., Todd et al., 2012). Nevertheless, the MMN elucidation does not depend on the subject’s active involvement and can be observed even in the fetus in the womb (e.g., Draganova et al., 2007) or in coma patients (see Morlet and Fischer, 2014 for a recent review). Because of its pre-attentive character the MMN is independent of fluctuations in vigilance and motivation which may be of special importance at long EEG recordings or in older and/or clinical populations (Näätänen et al., 2004).

Depending on the interval length between the standard and deviant tones (interstimulus interval, ISI), the MMN is suitable to determine two different, but strongly interrelated, processes. In case of a short ISI of 2 s or less (see Cheng et al., 2013), the MMN primarily reflects the detection of a mismatch between a stored auditory regularity and the current presentation of the environment and can therefore be considered as an index of the pre-attentive auditory discrimination ability (for a review see Näätänen et al., 2012). With increasing ISI, the MMN provides information on the duration of the pre-attentive auditory memory trace for the standard tone (for a review see Bartha-Doering et al., 2015). In young, healthy adults the auditory memory trace approximates 10 s (Böttcher-Gandor and Ullsperger, 1992; Sams et al., 1993; see Bartha-Doering et al., 2015 for a recent review on MMN in healthy and clinical populations).

A limited number of studies so far investigated the MMN in normal compared to pathological aging, specifically in AD, with equivocal results. While some studies report an attenuated MMN in AD for short (e.g., Schroeder et al., 1995) as well as for long ISIs (e.g., Pekkonen et al., 1994; Papadaniil et al., 2016), others failed to find MMN differences between AD and healthy controls (e.g., Kazmerski et al., 1997; Engeland et al., 2002; Brønnick et al., 2010; Hsiao et al., 2014). Studies investigating MMN in older adults with MCI are even scarcer. The majority of studies report an altered MMN in MCI in comparison to matched controls (e.g., Lindín et al., 2013; Ji et al., 2015; Papadaniil et al., 2016; but see Tsolaki et al., 2017 for contrary results). However, the results vary in MMN parameter (i.e., amplitude, latency), MMN localization (i.e., frontal, temporal), and the applied ISI length (i.e., short, long).

In sum, previous studies suggest that MMN after short as well as after long ISIs has the potential to be a biomarker for cognition, where the results for MMN after long ISIs are more consistent than for short ISIs. On the other hand, the MMN after short ISIs is the most often investigated one in AD research so far. Since the impact of pre-attentive auditory memory processes increases with ISI length, auditory memory processes seem to be an important factor that is responsible for the MMN-cognition relationship. We assumed that the difference score between MMN after short and long ISI (ΔMMN) might be a better and more reliable biomarker for cognitive (especially episodic memory) decline, compared to the simple MMN after long or short ISI, since ΔMMN takes individual differences in auditory discrimination ability as well as auditory memory into account. The difference between MMN after short and after long ISI is strongly determined by the pre-attentive maintenance of the memory trace for the standard tone. On the one hand ΔMMN would be 0 if the MMN amplitude after the short and long ISI is the same and thus the standard tone is well remembered independent of the ISI. On the other hand, the ΔMMN would become higher as the MMN amplitude attenuates as a function of the ISI. Thus, the ΔMMN could constitute a biomarker for automatic auditory memory decay, which in turn seems to be the key index for cognitive decline.

The main aim of the present study was to evaluate the validity of pre-attentive auditory memory decay as well as the MMN after short and long ISI as biomarkers for cognitive decline in an at risk population for AD (i.e., aMCI, naMCI, SMI). MMN after short ISI was assessed using the Optimum–1 (Opt1, stimulus onset asynchrony [SOA] = 0.5 s) paradigm (see Näätänen et al., 2004), whereas the MMN after long ISI (3 s) was investigated using the Memory Trace (MemTra) paradigm (in accordance with Grau et al., 1998). Pre-attentive memory trace decay was assessed by the difference score between the MMN after short and long ISIs (ΔMMN).

We hypothesized that pre-attentive auditory memory decay, reflected by the ΔMMN at baseline, is positively associated with episodic memory performance assessed at baseline as well as episodic memory 5 years later (5-year-follow-up). Further, we expected a smaller MMN in the MemTra paradigm in comparison to the Opt1 paradigm and a more pronounced decay of the pre-attentive auditory memory trace in aMCI compared to naMCI/SMI subjects.

## Materials and Methods

### Participants

The inclusion and exclusion criteria for study participation have previously been described in detail (Küster et al., 2016). In brief, subjects were recruited in the Memory Clinic of the University Hospital Ulm, Germany and the Center for Psychiatry Reichenau, Germany or via public advertisements. Inclusion criteria were: 55 years of age or older, fluency in the German language, subjective memory complaints or MCI, stable antidementive and/or antidepressive medication, normal or adjusted-to-normal hearing, and independent living. Exclusion criteria were: probable moderate or severe dementia (Mini-Mental State Examination, MMSE [Folstein et al., 1975] < 20), a history of other neurological or psychiatric disorders, except mild to moderate depression). Depressive symptoms were assessed with the 15-item short version of the Geriatric Depression Scale (Yesavage et al., 1983). Participants without contraindication were offered structural magnetic resonance imaging (MRI) to exclude other brain abnormalities such as major strokes and brain tumors.

SMI was assessed with the question “Do you feel like your memory is getting worse?” (according to Geerlings et al., 1999; Jessen et al., 2010). The evaluation of objective cognitive impairment was based on encoding (sum of words of the five learning trials) and long-delay free recall scores of the adapted German version of the California Verbal Learning Test (German: Münchner Verbaler Gedächtnistest [MVGT, Munich Vebal Memory Test]; Ilmberger, 1988) for memory functions. For non-memory cognitive functions the following subtests from the Consortium to Establish a Registry for Alzheimer’s Disease–plus (Welsh et al., 1994) were used: Trail Making Test (TMT) part A and B, phonematic and semantic word fluency, and Boston Naming Test. Objective cognitive impairment was defined as 1.0 *SD* below the age- and education-adjusted norm; aMCI was assigned if at least one of the memory tests was below average; naMCI was assigned if performance in the memory tests was average while one of the test scores of the other cognitive domains was below average. Subjects with severe objective impairment (≤ 2 *SD* below the norm) in memory and non-memory, indicating probable dementia, were excluded from further analysis (*n* = 6), even if they reached the critical MMSE score ≥ 20.

From altogether 122 subjects who were screened for eligibility, 59 met the inclusion criteria. For 14 participants no MRI scan was available. No participant had to be excluded because of abnormalities in the MRI scan. According to the classification criteria 16 subjects were classified as SMI, 19 as naMCI and 24 as aMCI. Demographic and cognitive characteristics of the groups are listed in Table 1. Groups did not differ with regard to distribution of gender or crystallized (premorbid) intelligence as indicated by a Verbal Knowledge Test (German: Wortschatztest; Schmidt and Metzler, 1992; all *p*s > 0.05). However, aMCI subjects showed lower education (*p* = 0.041) and tended to be older than naMCI and SMI which was, however, not significant (*p* = 0.098).

**Table 1:** Demographic and cognitive data within groups.

Variable	Group values			aMCI vs. naMCI/SMI	
	SMI (*n* = 14)	naMCI (*n* = 19)	aMCI (*n* = 24)	*F*_(1,55)_	*p*
Age (y.)	71.9 ± 5.4	68.1 ± 6.1	72.5 ± 6.2	2.83	0.098
Education (y.)	11.1 ± 1.9	10.5 ± 1.8	9.7 ± 1.8	4.37^a^	0.041
WST (*z*)	1.1 ± 0.8	0.6 ± 0.9	0.6 ± 0.7	0.89	0.349
MMSE (0–30)	28.9 ± 0.9	28.7 ± 1.2	27.5 ± 1.8	10.46	0.002
Episodic memory (cs)	0.6 ± 0.4	0.6 ± 0.7	−0.8 ± 0.5	92.15	<0.001
Attention/EF (cs)	0.6 ± 0.8	−0.1 ± 0.9	−0.3 ± 0.9	4.96	0.030
ADAS free rec. (0–10, error rate)	4.1 ± 1.4	4.8 ± 1.8	5.7 ± 1.4	8.29	0.006
Digit span (0–28)	15.0 ± 3.6	14.4 ± 3.6	13.6 ± 2.5	1.52	0.223
ECB (0–28)	14.7 ± 4.6	16.0 ± 5.0	10.9 ± 5.4	10.20^b^	0.002
MVGT enc. (0–80)	50.5 ± 5.4	52.4 ± 8.1	36.2 ± 5.1	81.57	<0.001
MVGT rec. (0–16)	10.9 ± 2.3	11.0 ± 2.5	4.6 ± 2.8	84.11^a^	<0.001
TMT A (s)	38.0 ± 8.4	53.8 ± 14.7	54.0 ± 19.7	2.32	0.134
TMT B (s)	92.2 ± 22.0	121.3 ± 46.2	129.8 ± 48.5	3.14	0.082
Word fluency (w.)	40.1 ± 7.8	31.3 ± 6.7	32.0 ± 7.8	1.65^a^	0.204

*Values are means (M) ± standard deviations (SD). aMCI, amnestic MCI; naMCI, non-amnestic MCI; SMI, subjective memory impairment; WST, Wortschatztest [Verbal Knowledge Test]; MMSE, Mini-Mental State Examination; ADAS free rec., Alzheimer’s Diseases Assessment Scale–free recall; Digit span, total value from the forward and backward part; ECB, Everyday Cognition Battery–computation span; MVGT enc., Münchner Verbaler Gedächtnistest [Munich Verbal Memory Test]–encoding (sum of words of the five learning trials); MVGT rec., Münchner Verbaler Gedächtnistest [Munich Verbal Memory Test]–long-delay free recall; TMT, Trail Making Test; Word fluency, total value of the episodic and phonemic word fluency; EF, executive functions; y., years; cs, composite score; w., words. Distribution of gender–aMCI vs. naMCI/SMI: ${\text{χ}}_{\text{(1)}}^{\text{2}}$ = 3.18, p = 0.074. ^a^F_(1,54)_; ^b^F_(1,50)_*.

### Procedure

The study was approved by the ethics committees of both study centers, University of Konstanz and Ulm University, Germany. The study was part of a controlled clinical trial investigating the effect of physical exercise and cognitive training on cognition as well as on biological and electrophysiological parameters (Küster et al., 2016, 2017; Fissler et al., 2017). All participants provided written informed consent in accordance with the Declaration of Helsinki prior to study participation. The neuropsychological assessment and the EEG examination were carried out by intensively trained assessors (i.e., doctorial and psychology students). Both MMN paradigms were carried out at the same session. Prior to the beginning of the EEG recordings, individual hearing thresholds were assessed using in-house software PyTuneSounds (Hartmann, 2009).

Five years (*M* = 5.23, *SD* = 0.19) after baseline assessment, a telephone interview-based follow-up was obtained for participants of the Konstanz sample. Out of the 32 potential follow-up participants, 28 subjects could be contacted again, five subjects refused participation and another two subjects had to be excluded since they were no longer able to attend the telephone interview due to severe progression of cognitive and functional decline. As a result, 21 complete data sets were available for follow-up analysis with four subjects classified as SMI, 11 as naMCI and six as aMCI at the baseline assessment. Because of the limited neuropsychological assessment no renewed classification was carried out at the 5-year-follow-up. All participants were asked if they had received an AD diagnosis during the past 5 years, which was not the case for the final *n* = 21.

### Neuropsychological Assessment

All participants completed the following assessments: the Alzheimer’s Disease Assessment Scale–cognitive subscale (Ihl and Weyer, 1993), phonemic and semantic word fluency as well as TMT part A and B of the Consortium to Establish a Registry for Alzheimer’s Disease–plus test battery, the subtests digit span and digit-symbol coding of the Wechsler Adult Intelligence Scale (Tewes, 1991), and the MVGT. Additionally, everyday cognition in an ecologically valid task was assessed using the working-memory subtest of the Everyday Cognition Battery (Allaire and Marsiske, 1999). Crystallized abilities were assessed using the Verbal Knowledge Test (German: Wortschatztest).

In order to assess latent cognitive function scores, a principal component analysis was performed across all participants (*n* = 59) to reduce multiple testing and thus α-inflation. An oblique rotation technique was chosen for the assumption of correlations between the extracted components. The following test scores were entered: MVGT encoding, MVGT free long-delay recall, free recall of the Alzheimer’s Disease Assessment Scale, TMT part A and B (time in sec), Everyday Cognition Battery–computation span, digit span forward and backward (total value), digit-symbol coding, and semantic and phonemic word fluency (total value as indicator for verbal word fluency). Using the Kaiser criterion (eigenvalues ≥ 1.0) two components were extracted, the first one showing high loadings of episodic memory scores (MVGT encoding, MVGT long-delay free recall, and free recall of the Alzheimer’s Disease Assessment Scale) and the second one showing high loadings of attention and executive functions scores (TMT part A and B, digit span, digit-symbol and verbal word fluency). All variables were *z*-standardized and two component scores were built, representing the weighted average of those *z-standardized* variables with loadings of at least *a*_ij_ = 0.50 on the respective component. Only the Everyday Cognition Battery–computation span did not reach the critical threshold (*a*_ij_ = 0.48) and was excluded from further component calculation.

For the follow-up investigation we selected tests from the baseline investigation, which were suitable for assessments via telephone (for telephone tools for cognitive assessment see e.g., Castanho et al., 2014; Duff et al., 2015), namely the MVGT, the digit span forward and backward, and the Consortium to Establish a Registry for Alzheimer’s Disease–plus subtests phonemic and semantic word fluency. The composite scores were built in the same manner as at baseline using the same weights from the available variables, i.e., MVGT encoding and MVGT long-delay free recall for the memory domain score; and digit span (total value for forward and backward) and verbal word fluency (total value for phonemic and semantic word fluency) for the attention/executive domain score.

### MMN Stimuli and Task Procedure

Two passive mismatch-negativity paradigms were applied: the Opt1 paradigm (see, Näätänen et al., 2004) to assess auditory discrimination ability and the newly developed MemTra paradigm (in accordance with Grau et al., 1998) to investigate auditory memory trace. The standard tone, duration and frequency deviant were further used in the MemTra paradigm (see below). The standard tone was a harmonic tone of three sinusoidal partials of 500, 1000 and 1500 Hz with the second partial being 3 dB and the third being 6 dB lower in intensity then the first partial. The standard tone was 75 ms in duration including 5 ms rise and fall times. In comparison to the standard tone, the duration deviant was 50 ms shorter and the gap deviant comprised a 7 ms silent gap (including 1 ms fall and rise times) in the middle of the tone. One half of all frequency deviants were 10% higher (partials: 550, 1100, 1650 Hz) and the other half 10% lower in frequency than the standard tone (partials: 450, 900, 1350 Hz). Intensity deviants were 10 dB louder or lower than the standard tone (50% each). The location deviants had an interaural time difference of 800 μs to the left or to the right ear (50% each).

### Optimum–1 Paradigm

**Figure 1:**
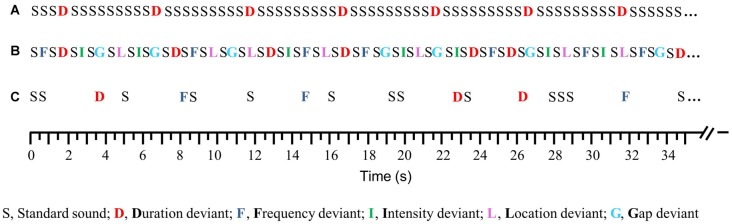
Schematic illustration of the auditory paradigms, compared to the traditional oddball paradigm, which was not used in the study. **(A)** Traditional oddball paradigm (SOA = 0.5 s), **(B)** Optimum–1 (Opt–1) paradigm (SOA = 0.5 s), and **(C)** Memory Trace (MemTra) paradigm (ISI = 0.5, 1.5, and 3 s; ISI_standard-deviant_ = 3 s).

In the Opt1 paradigm (Näätänen et al., 2004; Figure 1) a total number of 1845 auditory stimuli were presented in three blocks of 5 min each. Every second tone was a standard tone, resulting in a probability of 50% for standard and deviant stimuli and a probability of 10% for each deviant type. A sequence of 15 standard tones was presented at the beginning of each block to allow the formation of the standard tone as such. Stimuli were presented with a constant SOA of 0.5 s. Thus, the Opt1 paradigm is suitable to investigate the MMN after short ISI for five deviant types in a very short administration time.

### Memory Trace Paradigm

The MemTra paradigm (Grau et al., 1998; Figure 1) was developed to investigate the effect of longer ISIs on the MMN-related memory trace. The paradigm presented 462 auditory stimuli within three blocks of 6 min each. As in the Opt1 paradigm, 15 standard tones were presented consecutively at the beginning of each block. Duration and frequency deviants were presented with one to three standard tones between two deviants. The ISI between standard tone and consecutive deviant was constantly 3 s. The number of standard stimuli between the deviants and the ISI (0.5 s, 1.5 s and 3 s) between standard tones were assigned pseudorandomly. Standard stimuli were presented with 66.2% probability; deviants (duration, frequency) with the probability of 16.9% each. Only MMNs elicited to deviants following a standard tone (ISI = 3 s) were included into MMN analysis.

### EEG Recording

EEG was recorded using a high-density 256-channel HydroGel™ Geodesic Sensor Net (HCGSN; Electrical Geodesics, Inc., Eugene, OR, USA) with Cz (vertex) as reference during data acquisition. Continuous data were sampled with 1000 Hz and hardware filters were set to 0.1 Hz high-pass and 100 Hz low-pass. After recording, the data were imported into MATLAB (version 2015b; The MathWorks, 2015) and preprocessed using the FieldTrip toolbox (version 20151012; Oostenveld et al., 2011). During EEG recordings, participants were sitting comfortably in an electrically shielded and sound-attenuated room watching silent Charlie Chaplin videos. All auditory stimuli were presented binaurally through stereo headphones with 50 dB above the individual hearing threshold. All participants were instructed to watch the video carefully and not to pay attention to the delivered tones. The paradigm order was counter-balanced between subjects.

### MMN Analysis

For both the Opt1 and the MemTra paradigm, EEG data were band-pass filtered in the range of 1–35 Hz (24 dB/octave) and noisy channels were interpolated using the average method before rereferencing the data to the linked mastoids. Continuous data were further down-sampled to 250 Hz, segmented into epochs starting 100 ms before and ending 350 ms after stimulus onset and baseline-corrected (100-ms pre-stimulus time window). After manually rejecting artifact contaminated epochs, the remaining epochs were averaged for the standard tone and for each deviant type separately. On average, no more than 20% of the trials were excluded. Consequently, the following number of trials was left for averaging in the Opt1 paradigm (values are means ± standard deviations): 753 ± 53 for the standard stimulus, 152 ± 11 for the duration deviant, 151 ± 11 for the frequency deviant, 150 ± 11 for the intensity deviant, 150 ± 11 for the location deviant, and 149 ± 13 for the gap deviant. In the MemTra paradigm the reaction to standard stimulus was averaged over 83 ± 6 trials, for the duration deviant over 65 ± 5 trials, and for the frequency deviant over 65 ± 5 trials. Difference waveforms between the ERPs to the standard and to the deviant stimuli were carried out for each paradigm and deviant type, respectively. The MMN search window was determined within 100–250 ms, corresponding to previous studies on older adults with MCI (see Mowszowski et al., 2012, 2014; Ji et al., 2015). The MMN amplitude was defined as the mean voltage in a 40 ms time window centered at the peak of the grand-average waveform of each group (SMI, aMCI and naMCI). The MMN latency was defined at the most negative peak within the MMN search window after deviant onset (100–250 ms for frequency, intensity, and location deviants; 125–275 for duration deviant, and 134–284 ms for gap deviant). As the largest MMN is often assessed at fronto-central EEG electrodes and the averaging of electrodes with similar activity has been demonstrated to show more reliable results than the measure of single separate electrodes (Huffmeijer et al., 2014), the average voltage at FCz, Fz, and Cz was computed as mean MMN amplitude for all further analyses. The mean MMN latency was computed accordingly.

In two subjects (both SMI) the MMN amplitude extracted from the difference between standard and deviant tone showed a value above mean (−0.68 for duration and −0.17 frequency deviant) + 1.5 × interquartile range and >2 μV for both deviant types (duration and frequency) in the MemTra paradigm. Because of this abnormally high positive value (the difference score should be negative or around zero), we assumed that the paradigm did not work for them properly. To avoid any inaccuracies we excluded their datasets from all further analyses.

### Statistical Analyses

All statistical analyses were performed using R (version 3.2.3; R Core Team, 2016) in RStudio (RStudio Team, 2015). Statistical analyses of the baseline sample were performed with 57 subjects (24 classified as aMCI, 19 as naMCI and 14 as SMI). For one SMI subject only data for the Opt1 paradigm and for one aMCI subject only the MemTra paradigm data were available. All contrasts for group comparisons were set to aMCI vs. naMCI/SMI. Since all model residuals were normally distributed, parametric tests were applied for group comparisons. Comparisons for age, years of education, and cognitive function were conducted with univariate analysis of variance (ANOVA) models. Group differences in gender distribution were assessed by Pearson’s Chi-square (*χ*^2^)-test.

As a first step of the statistical ERP analysis, one-tailed *t*-tests for dependent samples for normally distributed data and Wilcoxon signed-rank tests for non-normally distributed data were conducted to determine whether mean MMN amplitudes significantly differed from zero within groups. Second, since both MMN paradigms applied a different number of deviants (five in the Opt1 and two in the MemTra paradigm), we explored differences in mean MMN amplitudes and latencies depending on group and deviant type for each paradigm separately.

The statistical models were carried out as follows: (1) for the MMN after short ISI (i.e., Opt1 paradigm) we conducted Group (aMCI vs. naMCI/SMI) × Deviant Type (duration, frequency, intensity, location, gap) linear mixed effect models with Subject as random intercept (lme4 package in R version 1.1-12; Bates et al., 2015) separately for mean MMN amplitude and mean MMN latency as dependent variable. (2) For the MMN after long ISI, statistical analyses focused only on the duration deviant, since there was no significant MMN component elicited by the frequency deviant in the MemTra paradigm. In order to investigate differences in the mean MMN amplitude for the duration deviant between paradigms (and thus ISIs), we carried out a Paradigm (Opt1/SOA = 0.5 s vs. MemTra/ISI = 3 s) × Group (aMCI vs. naMCI/SMI) linear mixed effects model including Subject as random intercept. (3) Second level (*post hoc*) analyses were conducted using univariate ANOVA models and pairwise *t*-tests with Bonferroni correction for multiple comparisons (multcomp package for R version 1.4-6; Hothorn et al., 2008).

As a final step, hierarchical linear regression models were carried out to investigate associations between MMN after short ISI, MMN after long ISI and pre-attentive auditory memory trace decay (i.e., ΔMMN for the duration deviant; ΔMMN–Dur) and neuropsychological composite scores for episodic memory and attention/executive functions at baseline (*n* = 57) and follow-up assessment (*n* = 21) as dependent variables, respectively. Based on previous research indicating higher age as a risk factor and education as a protective factor for cognitive decline (e.g., Ardila et al., 2000; Cansino, 2009; Salthouse, 2009, 2012), we statistically accounted for age and education by entering them into the model first (reduced models), followed by ΔMMN–Dur and MemTra MMN after duration deviants (MemTra MMN–Dur) as predictors in two separate models (full models). To explore the effect of Opt1 MMN we entered an Opt1 MMN × Deviant Type (duration, frequency, intensity, location, gap) term into the model instead of calculating models for each deviant type separately which would increase multiple testing and thus α-inflation. Using ANOVA, the full regression models were then compared to the reduced regression models without MMN indices as additional predictor. Collinearity between predictors was examined by computing the variance inflation factor (VIF) for each predictor’s beta score and for the mean beta score as well as the VIF tolerance score (1/VIF). Individual VIF scores > 10, a mean VIF score > 1, and a VIF tolerance score < 0.1 indicated beta score inflation by collinearity in the models (Bowerman and O’Connell, 1990; Myers, 1990; Menard, 1995). For illustration purposes significant associations between auditory memory and cognition are depicted as Pearson’s product-moment correlation coefficients (*r*).

Normal distribution of all models’ residuals was confirmed using the Shapiro-Wilk test (*W* statistic) and visual inspection (Q-Q plots). The statistical significance level (α) was set to 0.05 for all analyses.

The stability of significant associations between MMN indices and cognition was evaluated by the inclusion of the participants who were excluded before because of probable AD (see section “Participants”).

## Results

### ERP Analysis of the MMN

**Figure 2:**
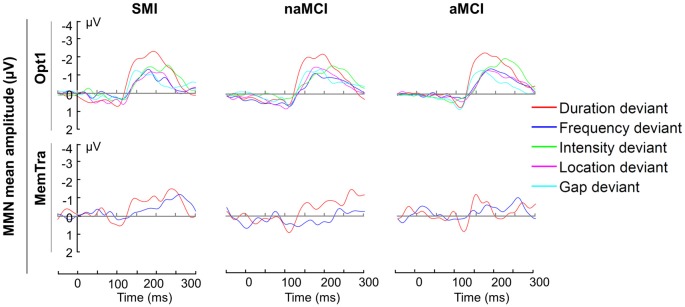
Mean MMN amplitudes for the Opt1 paradigm and MemTra paradigm within groups. Mean values are the averaged signal of the fronto-central electrodes Fz, FCz, and Cz. Opt1, Optimum–1; MemTra, Memory Trace; aMCI, amnestic MCI; naMCI, non-amnestic MCI; SMI, subjective memory impairment.

In order to examine whether all deviants elicited a MMN, one-tailed* t*-tests for dependent samples for normally distributed variables and Wilcoxon signed-rank tests for non-normally distributed data were conducted. Figure 2 shows the difference waveforms for the Opt1 and MemTra paradigm, respectively. The mean MMN difference waveform in the Opt1 condition significantly differed from zero for all deviant types in all groups (see Supplementary Table S1). In the MemTra paradigm only the difference waveform for the duration deviant significantly differed from zero in all groups (see Supplementary Table S1). Therefore, all further statistical analyses regarding the MemTra paradigm were restricted to the duration deviant (MMN–Dur).

### Group Differences between MMN Parameters

#### MMN after Short ISI

Analysis of the MMN after short ISI focused on the Opt1 paradigm and was conducted with linear mixed-effects models. The mixed-effects models with Group (aMCI vs. naMCI/SMI) as between-subject factor and Deviant Type (duration, frequency, intensity, location, gap) as within-subject factor showed a main effect of Deviant Type for both the mean MMN amplitude, *F*_(4,216)_ = 11.65, *p* < 0.001, and the mean MMN latency, *F*_(4,216)_ = 14.49, *p* < 0.001. Mean MMN amplitudes were largest for the duration deviant (*p*s ≤ 0.029) and mean MMN latencies were shortest for the duration and gap deviants (*p*s ≤ 0.131; *p*s ≤ 0.002, respectively).

Neither a main effect of Group nor a Group × Deviant Type interaction was found in both models (amplitude and latency; see Table 2 for MMN amplitudes and latencies for each group and see Supplementary Table S2 for pairwise comparison of the deviant types).

**Table 2:** Mean MMN amplitudes and latencies within groups.

MMN parameter	Group values			aMCI vs. naMCI/SMI	
	SMI	naMCI	aMCI	*F*_(1,54)_	*p*
		**Amplitudes**
Opt–Dur	−2.2 ± 1.1	−2.1 ± 1.2	−2.1 ± 1.1	0.01	0.910
Opt–Freq	−1.1 ± 0.7	−1.1 ± 1.0	−1.3 ± 1.0	1.02	0.317
Opt–Intens	−1.4 ± 1.0	−1.4 ± 1.3	−1.9 ± 1.4	1.76	0.191
Opt–Loc	−1.1 ± 0.8	−1.4 ± 1.6	−1.2 ± 1.1	0.01	0.935
Opt–Gap	−1.1 ± 1.3	−1.2 ± 1.0	−1.2 ± 1.0	0.02	0.880
MemTra–Dur	−1.1 ± 1.2	−1.0 ± 1.4	−0.5 ± 1.2	3.06	0.086
ΔMMN–Dur	−1.0 ± 1.3	−1.1 ± 1.4	−1.7 ± 1.2	3.03^a^	0.087
		**Latencies**
Opt–Dur	183.5 ± 23.5	184.5 ± 21.2	190.4 ± 29.8	0.74	0.394
Opt–Freq	191.7 ± 20.5	198.7 ± 21.7	199.0 ± 26.4	0.21	0.651
Opt–Intens	213.0 ± 23.9	208.5 ± 25.4	210.5 ± 29.7	0.50	0.483
Opt–Loc	195.0 ± 28.7	200.4 ± 27.7	199.6 ± 27.4	0.01	0.940
Opt–Gap	174.2 ± 20.6	184.9 ± 24.2	177.2 ± 28.0	0.01	0.913
MemTra–Dur	203.7 ± 34.1	184.4 ± 31.3	187.3 ± 31.9	1.92	0.172

*aMCI, amnestic MCI; naMCI, non-amnestic MCI; SMI, subjective memory impairment; Opt–Dur, MMN after duration deviants in the Optimum–1 paradigm; Opt–Freq, MMN after frequency deviants in the Optimum–1 paradigm; Opt–Intens, MMN after intensity deviants in the Optimum–1 paradigm; Opt–Loc, MMN after location deviants in the Optimum–1 paradigm; Opt–Gap, MMN after gap deviants in the Optimum–1 paradigm; MemTra–Dur, MMN after duration deviants in the Memory Trace paradigm; ΔMMN–Dur, difference score between MMN amplitude after duration deviant in the Optimum–1 paradigm and the Memory Trace paradigm, higher values indicating less auditory memory trace decay. ^a^F_(1,53)_*.

#### MMN after Long ISI and ISI Duration Effect

**Figure 3:**
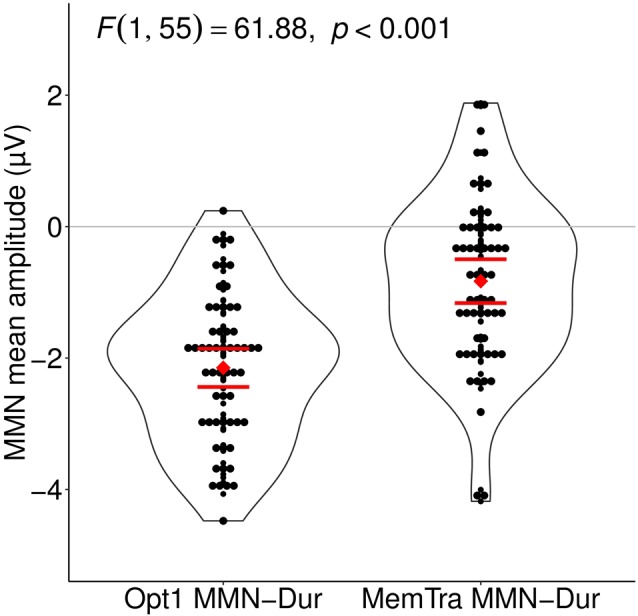
Mean MMN after duration deviants in comparison between the Opt1 and the MemTra paradigm. Mean values build from the averaged signal of the fronto-central electrodes Fz, FCz, and Cz. The depicted *F*-value is accounted for Group main effect and Group × Paradigm interaction. 95% confidence intervals for the average MMN amplitude are shown as horizontal bars, red dot representing the mean value. Opt1, Optimum–1; MemTra, Memory Trace; MMN Opt–Dur, MMN after duration deviants in the Optimum–1 paradigm; MMN Memtra–Dur, MMN after duration deviants in the Memory Trace paradigm.

Comparing the MMN amplitudes for the duration deviant between both paradigms, a mixed-effects model of Paradigm (Opt1 vs. MemTra) as within-subject factor × Group (aMCI vs. naMCI/SMI) as between-subject factor revealed a main effect of Paradigm, *F*_(1,55)_ = 61.88, *p* < 0.001, indicating smaller mean MMN–Dur amplitudes in the MemTra compared to the Opt1 paradigm (Figure 3), i.e., a stronger pre-attentive auditory memory decay in the long ISI condition. Subjects with aMCI showed a more pronounced pre-attentive auditory memory decay in comparison to naMCI and SMI, even though the Paradigm × Group interaction was only significant at a trend level, *F*_(1,55)_ = 3.21, *p* = 0.079 (see also Table 2). No main effect or interaction was found for the mean MMN–Dur latency.

### Associations between the MMN Parameters and Baseline Cognition

**Figure 4:**
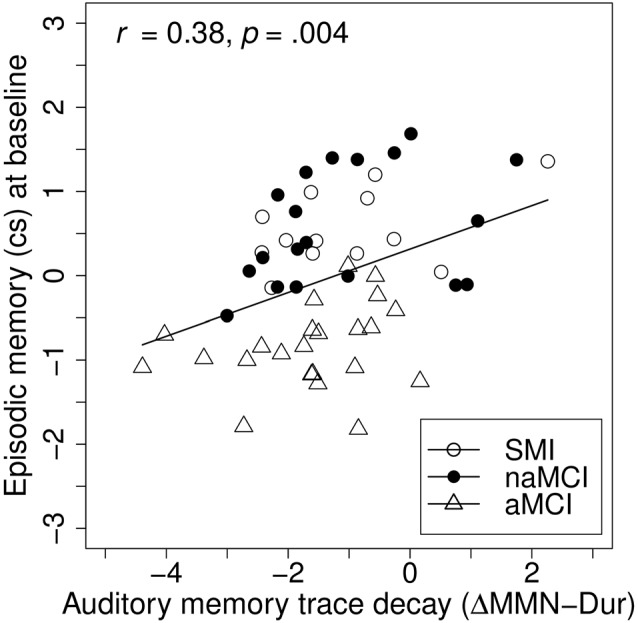
Baseline associations between auditory memory decay and episodic memory. Auditory memory reflected by the difference score between MMN amplitude after duration deviant in the Opt1 paradigm and the MemTra paradigm (ΔMMN–Dur), higher scores indicating less auditory memory trace decay. Opt1, Optimum–1; MemTra, Memory Trace, cs, composite score.

To investigate the associations between baseline MMN indices and baseline cognitive performance, linear hierarchical regression models were conducted across groups. The models were carried out separately for the episodic memory and the attention/executive functions composite scores as dependent variables, including age and education at baseline (reduced models) and additionally ΔMMN–Dur, MemTra MMN–Dur, or Opt1 MMN × Deviant Type term as predictors (full models; see Table 3). In the reduced models, age, but not education was a significant predictor of both episodic memory, *β* = −0.38, *t*_(50)_ = −2.94, *p* = 0.005, and attention/executive functions, *β* = −0.41, *t*_(50)_ = −3.30, *p* = 0.002, at baseline assessment; indicating worse performance in older participants. According to our hypothesis adding ΔMMN–Dur as predictor to the reduced model explained an additional 14% of the variance in the episodic memory score, *F*_(49,1)_ = 10.16, *p* = 0.002; *β*_ΔMMN–Dur_ = 0.38, *t*_(49)_ = 3.19, *p* = 0.002 (see also Figure 4 for correlative association). MemTra MMN–Dur as predictor explained an additional 7% of the variance in the episodic memory score, *F*_(50,1)_ = 4.14, *p* = 0.047; *β*_MemTra MMN–Dur_ = −0.27, *t*_(50)_ = −2.04, *p* = 0.047. There was no additive effect in predicting individual differences in the attention/executive functions score. No additive effect was found for the model including the Opt1 MMN × Deviant Type interaction.

**Table 3:** Baseline associations between auditory memory trace decay and episodic memory as well as executive functions accounting for age and education.

	Episodic memory				Attention/EF			
Predictor	*ΔR*^2^	*B*	*β*	*p*	*ΔR*^2^	*B*	*β*	*p*
Model 1	0.17			0.010	0.21			0.002
Age		−0.06	−0.38	0.005		−0.07	−0.41	0.002
Education		0.08	0.17	0.187		0.12	0.23	0.073
Model 2	0.14			0.002	0.02			0.214
Age		−0.05	−0.32	0.011		−0.06	−0.39	0.003
Education		0.07	0.16	0.183		0.11	0.22	0.078
ΔMMN–Dur		0.26	0.38	0.002		0.12	0.16	0.214

*EF, executive functions. ΔMMN–Dur, difference score between MMN amplitude after duration deviant in the Optimum–1 paradigm and the Memory Trace paradigm, higher values indicating less auditory memory trace decay*.

Even when the analysis was repeated with *n* = 6 subjects who were excluded because of probable AD the additive effect of ΔMMN–Dur remained significant, *F*_(54,1)_ = 13.93, *p* < 0.001; *β*_ΔMMN–Dur_ = 0.41, *t*_(54)_ = 3.73, *p* < 0.001.

### Associations between MMN Parameters and Cognition at the 5-Year-Follow-up

**Figure 5:**
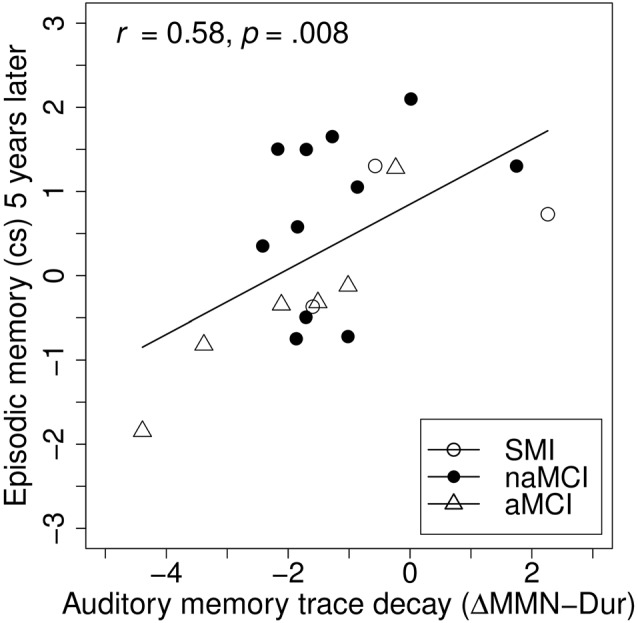
Associations between baseline auditory memory decay and follow-up episodic memory. Auditory memory reflected by the difference score between MMN amplitude after duration deviant in the Opt1 paradigm and the MemTra paradigm (ΔMMN–Dur), higher scores indicating less auditory memory trace decay. Opt1, Optimum–1; MemTra, Memory Trace, cs, composite score.

To investigate the prognostic effect of baseline auditory memory on cognition, linear hierarchical regression models were carried out across groups, separately for the episodic memory and the attention/executive functions composite score assessed 5 years later (see Table 4). In the reduced models including only age and education at baseline as predictors, neither age nor education was a significant predictor for episodic memory or attention/executive functions at the 5-year follow-up. Corroborating our hypothesis, including ΔMMN–Dur as additional predictor explained an additional 36% of the variance in the episodic memory (but not in the attention/executive functions) composite score compared to age and education entered alone, *F*_(16,1)_ = 7.91, *p* = 0.013; *β*_ΔMMN–Dur_ = 0.57, *t*_(16)_ = 2.81, *p* = 0.013 (see also Figure 5 for correlative association). No additive effects were found for MemTra MMN–Dur or the model including the Opt1 MMN × Deviant Type interaction. The additive effect of ΔMMN–Dur remained significant after inclusion of one subject (*n* = 6 at baseline) with probable AD, *F*_(17,1)_ = 8.63, *p* = 0.009; *β*_ΔMMN–Dur_ = 0.55, *t*_(17)_ = 2.94, *p* = 0.009.

**Table 4:** Associations between baseline auditory memory trace decay and follow-up episodic memory as well as executive functions accounting for age and education.

	Episodic memory				Attention/EF			
Predictor	*ΔR*^2^	*B*	*β*	*p*	*ΔR*^2^	*B*	*β*	*p*
Model 1	0.07			0.556	0.19			0.164
Age		−0.04	−0.25	0.294		−0.05	−0.39	0.090
Education		0.02	0.03	0.885		0.08	0.18	0.431
Model 2	0.31			0.013	0.01			0.592
Age		−0.03	−0.18	0.391		−0.04	−0.38	0.115
Education		0.07	0.11	0.596		0.09	0.19	0.405
ΔMMN–Dur		0.40	0.57	0.013		0.06	0.12	0.592

*EF, executive functions. ΔMMN–Dur, difference score between MMN amplitude after duration deviant in the Optimum–1 paradigm and the Memory Trace paradigm, higher values indicating less auditory memory trace decay*.

## Discussion

We investigated the auditory MMN after short and after long ISI as well as a novel pre-attentive auditory memory trace decay index in older adults with SMI, aMCI and naMCI as an at risk population of AD. The MMN after short ISI was investigated using the Opt1 paradigm (applying short ISI), with respect to five deviants (duration, frequency, intensity, location, gap). The MMN after long ISI was investigated using the MemTra paradigm (with long ISI) with respect to two different auditory deviant types (duration, frequency). Pre-attentive auditory memory trace decay was assessed by the difference score between the MMN after short and long ISI (ΔMMN). In line with the majority of studies (Verleger et al., 1992; Kazmerski et al., 1997; Gaeta et al., 1999; Brønnick et al., 2010; Cheng et al., 2012; Hsiao et al., 2014), we found no group differences in MMN after short ISI (see Pekkonen et al., 1994; Ruzzoli et al., 2016; but see also Engeland et al., 2002 for contrary results) suggesting preserved pre-attentive auditory encoding in MCI (aMCI and naMCI) in comparison to SMI. As a proof of concept, all groups showed an attenuated MMN in the MemTra (ISI = 3 s) in comparison to the Opt1 paradigm (SOA = 0.5 s) for the duration deviant.

In line with our main hypothesis, the ΔMMN–Dur (indicative of the pre-attentive auditory memory trace decay for the duration deviant) was positively associated with episodic memory performance across groups at baseline and 5 years later even after accounting for age and education. In contrast, no such relation was found for attention/executive functions, which is in line with previous work by Ruzzoli et al. (2012). The authors investigated the MMN for duration deviants in an auditory oddball paradigm with 4 s ISI in a healthy adult sample aged 21–60 years. In this, the frontal MMN for duration deviants was positively correlated with memory performance but not with executive functions. Foster et al. (2013) reported a positive association between MMN employing different ISIs as standard and deviants and verbal memory assessed with the Rey Auditory Verbal Learning Test in older healthy adults.

Shared underlying neurobiological mechanisms might be responsible for the association between pre-attentive auditory memory trace decay and episodic memory. Interestingly, the pre-attentive auditory memory trace, measured with the MMN, especially after long ISIs, and episodic memory are both related to N-methyl-D-aspartate (NMDA) receptor functioning. It is well known that the NMDA receptor is highly involved in neuronal plasticity, long-term-potentiation, as well as learning and episodic memory (for a review see Newcomer et al., 2000). A distorted NMDA receptor-subunit expression and functionality has been reported for healthy older adults and in AD (Mishizen-Eberz et al., 2004; Amada et al., 2005; for a review see Magnusson et al., 2010) and is thought to be involved in age-associated cognitive impairment (for a review see Kumar, 2015). Recently, the decay in the pre-attentive auditory memory trace has also been discussed in the context of NMDA receptor modulation of plasticity, and predictive coding theory (Friston, 2005; Garrido et al., 2009; Näätänen et al., 2014), an integrative model to explain the formation of the MMN within the fronto-temporal network (Friston, 2005; Baldeweg, 2006). Predictive coding considers neuronal activity as a reflection of matches or mismatches between internal predictions based on previous experiences stored in short-term memory and current external events (Heekeren et al., 2008). The theory of predictive coding is well studied in the visual domain (see Stefanics et al., 2014 for a recent review) and has also been increasingly discussed for auditory processing in recent years (e.g., Friston, 2005; Garrido et al., 2009). Regarding the auditory paradigms used in this study, this can be a form of a detection error, indexed by the MMN, whenever the incoming information (deviant tone) does not match the prediction (standard tone). The memory trace formation for the standard tone as well as its changes demand short-term synaptic plasticity which is codetermined by an intact NMDA receptor activity (e.g., Garrido et al., 2009).

The MMN after the duration deviant was significantly larger than the one after the other four deviant types (see also Figure 2) in the Opt1 paradigm. Näätänen et al. (2004) report the same finding in healthy young adults. Thus, it seems as if the fronto-temporal network described above is more sensitive to deviations in duration in comparison to deviations in frequency, intensity, location, or a gap in the middle of the tone.

No MMN was detectable in the MemTra paradigm for the frequency deviant, which might indicate that the slope of memory trace decay varies for different tone characteristics (in case of the MemTra paradigm duration and frequency), with the memory trace for frequency deviants fading faster with time compared to duration deviants. Consequently, our results regarding the applicability of ΔMMN are restricted to MMN for duration deviants. To our best knowledge, no study exists to date which investigated MMN after different deviant types and for different ISI lengths within one AD or MCI sample. Contrary to our results, two studies investigating the MMN after duration and frequency deviants for short as well as long ISIs in healthy older adults indicate a faster decay of the pre-attentive auditory memory trace for duration in comparison to frequency deviants (Schroeder et al., 1995; Pekkonen et al., 1996). However, Cooper et al. (2006) failed to find such differences in healthy aging. Interestingly, MMN for duration deviants suggests the best prognostic value in the prediction of psychosis in at risk individuals in comparison to frequency and intensity deviants (see Näätänen et al., 2015 for a recent review and Erickson et al., 2016 for a recent meta-analysis). Notably, the vast majority of studies of MMN in schizophrenia use short ISIs only.

Regarding the fact that subjects with aMCI have the highest risk to develop AD, we expected a more pronounced pre-attentive auditory memory trace decay reflected by the ΔMMN–Dur in aMCI in comparison to naMCI/SMI. This effect was only present at the trend level (*p* = 0.079). Nevertheless, visual inspections indicate a smaller MMN after long ISIs in aMCI compared to the other two groups (Figure 2).

It needs to be mentioned that aMCI subjects had a significantly lower education (*p* = 0.041; Table 1) in comparison to SMI and naMCI. This finding is in line with the well-studied findings of education as a protective factor against cognitive decline (e.g., Salthouse, 2009).

The following limitations need to be considered for this study: as all participants investigated in this study showed subjective or objective cognitive impairment, our results are restricted to this at risk of developing AD group only. Thus, we cannot draw any conclusion about the prognostic value of ΔMMN–Dur in healthy aging. The sample size of the study, especially in the follow-up investigation, was rather small. Nevertheless, we found hypothesis-confirming significant positive associations between auditory memory trace decay and episodic memory. As all tests included in the episodic memory composite score were verbal in nature (Alzheimer’s Disease Assessment Scale free recall, MVGT), it remains open whether the association between auditory memory and episodic memory is restricted to verbal memory only or if it can be generalized to other memory modalities.

Due to logistic reasons, we had two dropouts from the 5-year-follow-up due to severe cognitive and functional decline, a group of especially great interest. Larger sample sizes in future studies would help to handle dropout analyses. Future studies with larger sample sizes are needed to replicate the effects (including e.g., survival analyses).

## Conclusion

The strong significant association between ΔMMN–Dur and episodic memory at baseline and at the 5-year-follow-up provides an additional insight into neurobiological processes associated with pathological aging and may help in developing new tools for early diagnosis as well as for treatment monitoring. Since EEG recording is a non-invasive and cost-efficient tool, ΔMMN–Dur might become a useful extension to complement neuropsychological assessment in older populations at risk of developing AD. Further research and longitudinal studies with larger sample sizes and healthy age-matched as well as younger healthy controls are needed to evaluate possible clinical implications.

## Author Contributions

DL contributed to study conception and design, organized study procedures and acquired data, analyzed and interpreted the data, and wrote the first draft of the manuscript. FT contributed to study conception and design, organized study procedures, acquired data, contributed to data analysis, and critically revised the first draft of the manuscript and the article. PF and OCK contributed to study conception and design, organized study procedures and acquired data, contributed to data interpretation and critically revised the manuscript. SK supervised the statistical analysis of the data and critically revised the manuscript. WS provided support in data analyses and data interpretation and critically revised the manuscript. CAFA and I-TK conceptualized the study, obtained funding, supervised all phases of the study as principal investigators and critically revised the manuscript. All authors read and approved the final manuscript.

## Conflict of Interest Statement

The authors declare that the research was conducted in the absence of any commercial or financial relationships that could be construed as a potential conflict of interest.

## Abbreviations

AD: Alzheimer’s dementia
aMCI: amnestic mild cognitive impairment
MCI: mild cognitive impairment
MemTra: Memory Trace
MMN: mismatch negativity
MMN–Dur: mismatch negativity after duration deviants
MMSE: Mini-Mental State Examination
MVGT: Münchner Verbaler Gedächtnistest
naMCI: non-amnestic mild cognitive impairment
NMDA: N-methyl-D-aspartate
Opt1: Optimum–1
SMI: subjective memory impairment
TMT: Trail Making Test
ΔMMN: difference score between MMNs to short and to long ISIs
ΔMMN–Dur: index for auditory memory trace decay, amplitude difference between the MMN after duration deviant in the Optimum–1 paradigm and the Memory Trace paradigm, higher values indicating less auditory memory trace decay.
