# Supplementary material for: Auditory Memory Decay as Reflected by a New Mismatch Negativity Score Is Associated with Episodic Memory in Older Adults at Risk of Dementia
Source: Front Aging Neurosci. 2018 Feb 2;10:5. doi: 10.3389/fnagi.2018.00005 (PMC5801314; doi:10.3389/fnagi.2018.00005)
Supplement: Supplementary file 1 [file Table_1.docx]

Supplementary Material

**Auditory memory decay as reflected by a new mismatch negativity score is associated with episodic memory in older adults at risk of dementia**

Daria Laptinskaya^*^, Franka Thurm, Olivia Caroline Küster, Patrick Fissler, Winfried Schlee, Stephan Kolassa, Christine A. F. von Arnim, & Iris-Tatjana Kolassa

*** Correspondence:** Daria Laptinskaya: [daria.laptinskaya@uni-ulm.de](mailto:daria.laptinskaya@uni-ulm.de)

| **Supplementary Table 1. Significant difference wave at fronto-central area in the MMN search window measured by means of one-tailed *t*-tests if the variable was normal distributed and Wilcoxon signed-rank test if the variable was non-normally distributed.** | | | | | | | | | | |
| --- | --- | --- | --- | --- | --- | --- | --- | --- | --- | --- |
|  |  |  | **SMI** | |  | **naMCI** | |  | **aMCI** | |
|  |  |  | Statistic | *p* |  | Statistic | *p* |  | Statistic | *p* |
| Opt1-Dur | Fz |  | -6.76 ^a^ | <0.001 |  | -6.92 ^c^ | <0.001 |  | -8.40 ^d^ | <0.001 |
|  | FCz |  | -6.97 ^a^ | <0.001 |  | -7.37 ^c^ | <0.001 |  | -8.37 ^d^ | <0.001 |
|  | Cz |  | -6.19 ^a^ | <0.001 |  | -7.03 ^c^ | <0.001 |  | -10.31 ^d^ | <0.001 |
|  | Mean |  | -7.45 ^a^ | <0.001 |  | -7.61 ^c^ | <0.001 |  | -9.36 ^d^ | <0.001 |
| Opt1-Freq | Fz |  | -4.65 ^a^ | <0.001 |  | -3.68 ^c^ | 0.001 |  | -6.14 ^d^ | <0.001 |
|  | FCz |  | -5.77 ^a^ | <0.001 |  | -4.56 ^c^ | <0.001 |  | -6.33 ^d^ | <0.001 |
|  | Cz |  | -5.13 ^a^ | <0.001 |  | -5.23 ^c^ | <0.001 |  | *V* = 0 | <0.001 |
|  | Mean |  | -5.34 ^a^ | <0.001 |  | -4.58 ^c^ | <0.001 |  | -6.66 ^d^ | <0.001 |
| Opt1-Intens | Fz |  | *V* = 0 | <0.001 |  | -4.36 ^c^ | <0.001 |  | -6.38 ^d^ | <0.001 |
|  | FCz |  | -4.76 ^a^ | <0.001 |  | *V* = 1 | <0.001 |  | -6.50 ^d^ | <0.001 |
|  | Cz |  | -5.83 ^a^ | <0.001 |  | *V* = 0 | <0.001 |  | -5.89 ^d^ | <0.001 |
|  | Mean |  | *V* = 0 | <0.001 |  | *V* = 1 | <0.001 |  | -6.37 ^d^ | <0.001 |
| Opt1-Loc | Fz |  | *V* = 2 | <0.001 |  | -3.86 ^c^ | 0.001 |  | -5.72 ^d^ | <0.001 |
|  | FCz |  | -5.14 ^a^ | <0.001 |  | -3.84 ^c^ | 0.001 |  | -5.61 ^d^ | <0.001 |
|  | Cz |  | -5.20 ^a^ | <0.001 |  | -3.99 ^c^ | <0.001 |  | -5.17 ^d^ | <0.001 |
|  | Mean |  | *V* = 0 | <0.001 |  | -3.94 ^c^ | <0.001 |  | -5.63 ^d^ | <0.001 |
| Opt1-Gap | Fz |  | -3.27 ^a^ | 0.003 |  | -4.88 ^c^ | <0.001 |  | -5.31 ^d^ | 0.001 |
|  | FCz |  | -3.16 ^a^ | 0.004 |  | -5.36 ^c^ | <0.001 |  | -6.04 ^d^ | <0.001 |
|  | Cz |  | -3.12 ^a^ | 0.004 |  | *V* = 3 | <0.001 |  | -5.89 ^d^ | <0.001 |
|  | Mean |  | -3.26 ^a^ | 0.003 |  | -5.10 ^c^ | <0.001 |  | -5.85 ^d^ | <0.001 |
| MemTra-Dur | Fz |  | -2.78 ^b^ | 0.008 |  | -3.16 ^c^ | 0.003 |  | -2.60 ^e^ | 0.008 |
|  | FCz |  | -2.37 ^b^ | 0.018 |  | -2.34 ^c^ | 0.016 |  | -2.04 ^e^ | 0.026 |
|  | Cz |  | *V* = 4 | <0.001 |  | -2.50 ^c^ | 0.011 |  | -1.00 ^e^ | 0.165 |
|  | Mean |  | -3.52 ^b^ | 0.002 |  | -3.25 ^c^ | 0.002 |  | -1.99 ^e^ | 0.030 |
| MemTra-Freq | Fz |  | -0.70 ^b^ | 0.249 |  | -0.99 ^c^ | 0.168 |  | -0.31 ^e^ | 0.381 |
|  | FCz |  | -1.52 ^b^ | 0.077 |  | -0.96 ^c^ | 0.175 |  | -0.93 ^e^ | 0.182 |
|  | Cz |  | *V* = 28 | 0.122 |  | 0.31 ^c^ | 0.619 |  | -1.06 ^e^ | 0.150 |
|  | Mean |  | -1.31 ^b^ | 0.107 |  | -0.63 ^c^ | 0.269 |  | -0.87 ^e^ | 0.197 |
| *The MMN area of interest was defined as the mean voltage of 40 µV centered at the peak latency of the most negative peak of the group grand average difference wave in the MMN search window of 100-250 ms after deviant onset (100-250 ms for frequency, intensity, and location; 125-275 for duration; and 134-284 ms for gap deviant). The values were tested against zero. Mean MMN is the average voltage of the signal from the fronto-central electrodes Fz, FCz, and Cz.*  *aMCI, amnestic MCI; naMCI, non-amnestic MCI; SMI, subjective memory impairment; Opt1-Dur, MMN after duration deviants in the Optimum*–*1 paradigm; Opt1-Freq, MMN after frequency deviants in the Optimum-1 paradigm; Opt1-Intens, MMN after intensity deviants in the Optimum*–*1 paradigm; Opt1-Loc, MMN after location deviants in the Optimum*–*1 paradigm; Opt1-Gap, MMN after gap deviants in the Optimum*–*1 paradigm; MemTra-Dur, MMN after duration deviants in the Memory Trace paradigm; MemTra-Freq, MMN after frequency deviants in the Memory Trace paradigm.*  ^a^ *t*(13); ^b^ *t*(12); ^c^ *t*(18); ^d^ *t*(22); ^e^ *t*(23). | | | | | | | | | | |
