# Supplementary material for: Auditory Memory Decay as Reflected by a New Mismatch Negativity Score Is Associated with Episodic Memory in Older Adults at Risk of Dementia
Source: Front Aging Neurosci. 2018 Feb 2;10:5. doi: 10.3389/fnagi.2018.00005 (PMC5801314; doi:10.3389/fnagi.2018.00005)
Supplement: Supplementary file 2 [file Table_2.docx]

Supplementary Material

**Auditory memory decay as reflected by a new mismatch negativity score is associated with episodic memory in older adults at risk of dementia**

Daria Laptinskaya^*^, Franka Thurm, Olivia Caroline Küster, Patrick Fissler, Winfried Schlee, Stephan Kolassa, Christine A. F. von Arnim, & Iris-Tatjana Kolassa

*** Correspondence:** Daria Laptinskaya: [daria.laptinskaya@uni-ulm.de](mailto:daria.laptinskaya@uni-ulm.de)

| **Supplementary Table 2. Pairwise comparisons for differences in amplitudes as well as latencies in the Optimum**–**1 (Opt1) paradigm after different deviant types.** | | | | | | |
| --- | --- | --- | --- | --- | --- | --- |
|  |  | **Amplitudes** | |  | **Latencies** | |
|  |  | *t*(55) | *p* |  | *t*(55) | *p* |
| Dur – Freq |  | 6.24 | <0.001 |  | 2.57 | 0.131 |
| Dur – Intens |  | 3.12 | 0.029 |  | 5.68 | <0.001 |
| Dur – Loc |  | 5.93 | <0.001 |  | 2.62 | 0.112 |
| Dur – Gap |  | 6.66 | <0.001 |  | -2.08 | 0.419 |
| Freq – Intens |  | -2.34 | 0.227 |  | 2.82 | 0.066 |
| Freq – Loc |  | -0.46 | >0.999 |  | 0.40 | >0.999 |
| Freq – Gap |  | -0.12 | >0.999 |  | -4.60 | <0.001 |
| Intens – Loc |  | 1.69 | 0.964 |  | -2.46 | 0.172 |
| Intens – Gap |  | 2.24 | 0.291 |  | -6.84 | <0.001 |
| Loc – Gap |  | 0.41 | >0.999 |  | -4.01 | 0.002 |
| *Since all differences (except the Freq*–*Loc comparison) were normally distributed, comparisons were carried out by pairwise t-tests using Bonferroni correction. Dur, duration; Freq, frequency; Intens, intensity; Loc, location.* | | | | | | |
